# Crystal structure of 4-azido­methyl-6-isopropyl-2*H*-chromen-2-one

**DOI:** 10.1107/S2056989015004387

**Published:** 2015-03-07

**Authors:** M. S. Krishnamurthy, Noor Shahina Begum, D. Shamala, K. Shivashankar

**Affiliations:** a Department of Studies in Chemistry, Central College Campus, Bangalore University, Bangalore 560 001, Karnataka, India

**Keywords:** crystal structure, 2*H*-chromen-2-one, π–π inter­actions, hydrogen bonding

## Abstract

In the title mol­ecule, C_13_H_13_N_3_O_2_, the benzo­pyran ring system is essentially planar, with a maximum deviation of 0.017 (1) Å. In the crystal, weak C—H⋯O hydrogen bonds link mol­ecules into ladders along [010]. In addition, π–π inter­actions between inversion-related mol­ecules, with centroid–centroid distances in the range 3.679 (2)–3.876 (2) Å, complete a two-dimensional network parallel to (001).

## Related literature   

For therapeutic properties of coumarin derivatives, see: Lacy & O’Kennedy (2004 ▸); Mustafa *et al.* (2011 ▸). For the biological activity of 2*H*-chromen-2-ones, see: Naik *et al.* (2012 ▸). For applications of organic azides, see: Kusanur *et al.* (2010 ▸). For structural features of coumarins, see: Moorthy *et al.* (2003 ▸). For related structures, see: Gowda *et al.* (2010 ▸); Fun *et al.* (2011 ▸); Nagarajaiah *et al.* (2013 ▸).
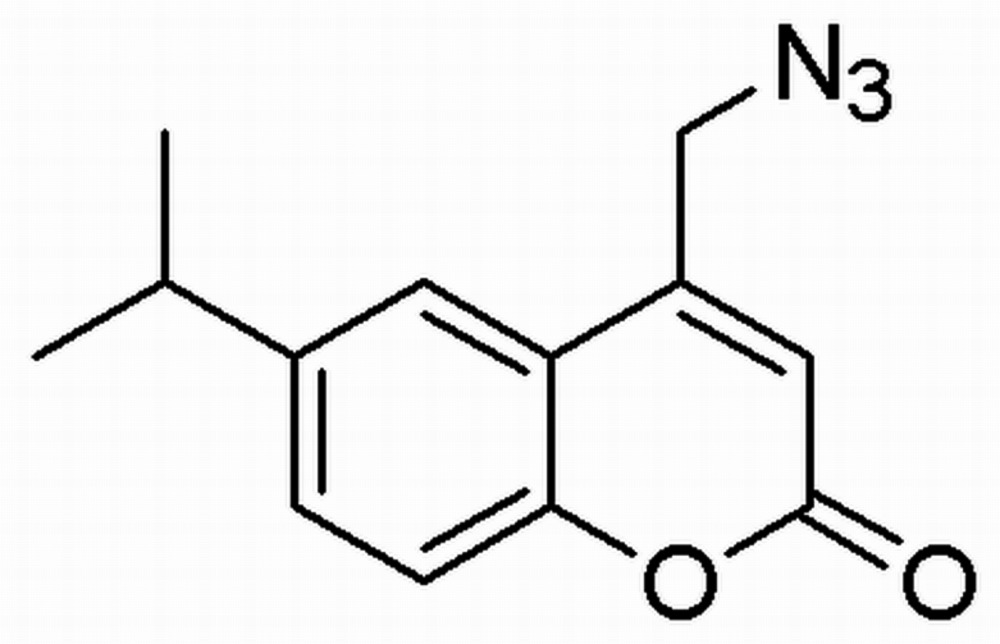


## Experimental   

### Crystal data  

C_13_H_13_N_3_O_2_
*M* *_r_* = 243.26
Triclinic, 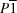

*a* = 6.895 (2) Å
*b* = 7.862 (2) Å
*c* = 11.592 (4) Å
α = 72.218 (6)°
β = 79.662 (5)°
γ = 82.430 (6)°
*V* = 586.7 (3) Å^3^
*Z* = 2
Mo *K*α radiation
μ = 0.10 mm^−1^
*T* = 100 K
0.18 × 0.16 × 0.16 mm

### Data collection  

Bruker SMART APEX CCD detector diffractometer
Absorption correction: multi-scan (*SADABS*; Bruker, 1998 ▸) *T*_min_ = 0.983, *T*_max_ = 0.985
3059 measured reflections
2026 independent reflections
1644 reflections with *I* > 2σ(*I*)
*R*_int_ = 0.013

### Refinement  

*R*[*F* ^2^ > 2σ(*F* ^2^)] = 0.051
*wR*(*F* ^2^) = 0.150
*S* = 1.07
2026 reflections
165 parameters
H-atom parameters constrained
Δρ_max_ = 0.34 e Å^−3^
Δρ_min_ = −0.26 e Å^−3^

### 

Data collection: *SMART* (Bruker, 1998 ▸); cell refinement: *SAINT* (Bruker, 1998 ▸); data reduction: *SAINT*; program(s) used to solve structure: *SHELXS97* (Sheldrick, 2008 ▸); program(s) used to refine structure: *SHELXL2014* (Sheldrick, 2015 ▸); molecular graphics: *ORTEP-3 for Windows* (Farrugia, 2012 ▸) and *PLATON* (Spek, 2009 ▸); software used to prepare material for publication: *WinGX* (Farrugia, 2012 ▸).

## Supplementary Material

Crystal structure: contains datablock(s) global, I. DOI: 10.1107/S2056989015004387/lh5754sup1.cif

Structure factors: contains datablock(s) I. DOI: 10.1107/S2056989015004387/lh5754Isup2.hkl

Supporting information file. DOI: 10.1107/S2056989015004387/lh5754Isup3.cml

**Fig. 1.**
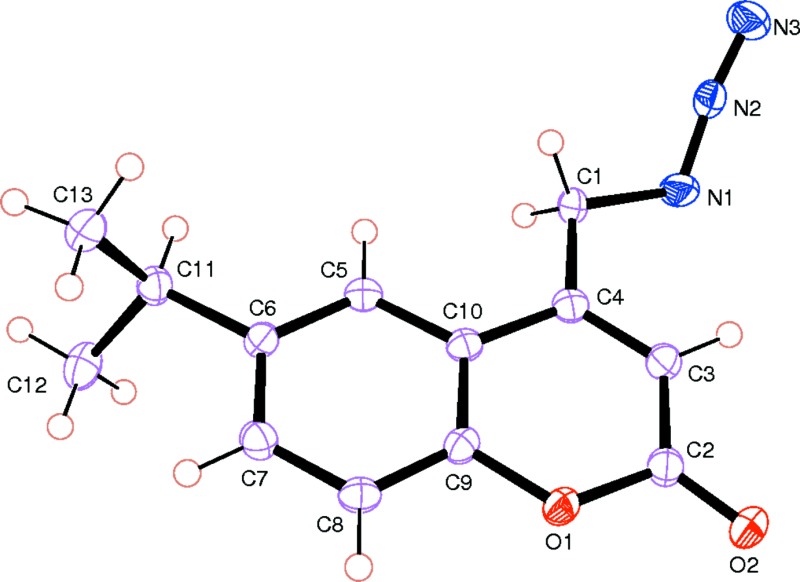
The molecular structure of the title compound. Displacement ellipsoids are drawn at the 50% probability level. H atoms are presented as small spheres of arbitrary radius.

. DOI: 10.1107/S2056989015004387/lh5754fig1.tif
The mol­ecular structure of the title compound. Displacement ellipsoids are drawn at the 50% probability level. H atoms are presented as small spheres of arbitrary radius.

**Fig. 2.**
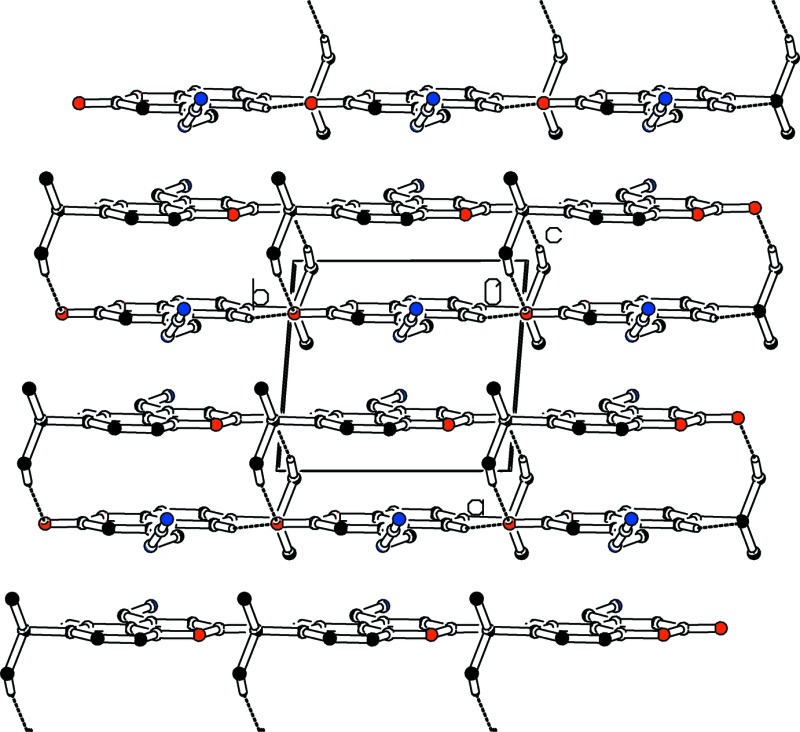
Part of the crystal structure with weak hydrogen bonds shown as dashed lines. Only H atoms involved in hydrogen bonds are shown.

. DOI: 10.1107/S2056989015004387/lh5754fig2.tif
Part of the crystal structure with weak hydrogen bonds shown as dashed lines. Only H atoms involved in hydrogen bonds are shown.

CCDC reference: 1051846

Additional supporting information:  crystallographic information; 3D view; checkCIF report

## Figures and Tables

**Table 1:** Hydrogen-bond geometry (, )

*D*H*A*	*D*H	H*A*	*D* *A*	*D*H*A*
C5H5O2^i^	0.95	2.56	3.498(2)	168
C13H13*C*O2^ii^	0.98	2.55	3.524(3)	172

Symmetry codes: (i) 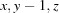; (ii) 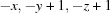.

## supplementary crystallographic information

### S1. Comment

Coumarins are of great interest due to their biological properties (Lacy & Kennedy 2004). In particular, their physiological, bacteriostatic and anti- tumour activity (Mustafa *et al.*, 2011) makes these compounds attractive for further backbone derivatization and screening for their therapeutic properties. In view of their extensive natural occurrence and biocompatibility, 2*H*-chromen-2-ones have been found to exhibit variety of biological activities (Naik *et al.*, 2012). In addition, organic azides are an important class of 1,3-dipoles, which have been recently recognized as crucial functional groups in click chemistry (Kusanur *et al.*, 2010). In view of the above, the title compound was isolated and the crystal structure is presented herein.

In the title compound (Fig. 1), the benzopyran ring system is essentially planar with a maximum deviation of 0.017 (1)Å for atom C10. Atom N1 of the azido group deviates by 0.168 (2)Å from the mean-plane of the benzopyran ring system while atoms N2 and N3, deviate by -0.489 (1) and -1.013 (2)Å, respectively from the opposite face of this ring system. In the crystal, weak C—H···O hydrogen bonds link molecules into ladders along [010] (Table 1, Fig.2). In addition, π–π interactions between inversion related molecules, with centroid–centroid distances in the range 3.679 (2)–3.876 (2)Å, complete a two-dimensional network parallel to (001).

### S2. Experimental

4-Bromomethyl-6-isopropylcoumarin (0.01 mol) was taken in acetone (20 ml) in a round bottomed flask. To this, sodium azide (0.012 mol, 0.78 g) in 5 ml of water was added drop wise with stirring for 3 hrs (reaction was monitored by TLC). The reaction mixture was poured into ice cold water, separated solid was filtered and recrystallized from ethyl acetate. (Yield 85%; colorless solid; mp 360 K).

### S3. Refinement

H atoms were placed in calculated positions in a riding-model approximation with C—H = 0.95, 0.98 and 0.99Å for aromatic, methyl and methylene H-atoms respectively, with *U*_iso_(H) = 1.5*U*_eq_(C) for methyl H atoms and *U*_iso_(H) = 1.2*U*_eq_(C) for other hydrogen atoms.

### Crystal data

**Table:**

C_13_H_13_N_3_O_2_	*Z* = 2
*M**_r_* = 243.26	*F*(000) = 256
Triclinic, *P*1	*D*_x_ = 1.377 Mg m^−^^3^
*a* = 6.895 (2) Å	Mo *K*α radiation, λ = 0.71073 Å
*b* = 7.862 (2) Å	Cell parameters from 2028 reflections
*c* = 11.592 (4) Å	θ = 1.9–25.0°
α = 72.218 (6)°	µ = 0.10 mm^−^^1^
β = 79.662 (5)°	*T* = 100 K
γ = 82.430 (6)°	Block, colourless
*V* = 586.7 (3) Å^3^	0.18 × 0.16 × 0.16 mm

### Data collection

**Table:**

Bruker SMART APEX CCD detector diffractometer	1644 reflections with *I* > 2σ(*I*)
Radiation source: fine-focus sealed tube	*R*_int_ = 0.013
ω scans	θ_max_ = 25.0°, θ_min_ = 1.9°
Absorption correction: multi-scan (*SADABS*; Bruker, 1998)	*h* = −8→7
*T*_min_ = 0.983, *T*_max_ = 0.985	*k* = −9→8
3059 measured reflections	*l* = −13→9
2026 independent reflections	

### Refinement

**Table:**

Refinement on *F*^2^	0 restraints
Least-squares matrix: full	Hydrogen site location: inferred from neighbouring sites
*R*[*F*^2^ > 2σ(*F*^2^)] = 0.051	H-atom parameters constrained
*wR*(*F*^2^) = 0.150	*w* = 1/[σ^2^(*F*_o_^2^) + (0.0929*P*)^2^ + 0.0624*P*] where *P* = (*F*_o_^2^ + 2*F*_c_^2^)/3
*S* = 1.07	(Δ/σ)_max_ < 0.001
2026 reflections	Δρ_max_ = 0.34 e Å^−^^3^
165 parameters	Δρ_min_ = −0.26 e Å^−^^3^

### Special details

**Table:**

Geometry. All esds (except the esd in the dihedral angle between two l.s. planes) are estimated using the full covariance matrix. The cell esds are taken into account individually in the estimation of esds in distances, angles and torsion angles; correlations between esds in cell parameters are only used when they are defined by crystal symmetry. An approximate (isotropic) treatment of cell esds is used for estimating esds involving l.s. planes.

### Fractional atomic coordinates and isotropic or equivalent isotropic displacement parameters (Å^2^)

**Table:**

	*x*	*y*	*z*	*U*_iso_*/*U*_eq_	
O1	0.21989 (18)	0.72869 (15)	0.55787 (11)	0.0218 (4)	
O2	0.25015 (19)	0.97173 (16)	0.40014 (12)	0.0275 (4)	
N1	0.3566 (3)	0.5078 (2)	0.18653 (14)	0.0295 (4)	
N2	0.2838 (2)	0.46732 (19)	0.11032 (14)	0.0224 (4)	
N3	0.2296 (3)	0.4459 (2)	0.02953 (15)	0.0347 (5)	
C1	0.3110 (3)	0.3983 (2)	0.31451 (15)	0.0208 (4)	
H1A	0.4222	0.3069	0.3361	0.025*	
H1B	0.1911	0.3354	0.3245	0.025*	
C2	0.2481 (3)	0.8106 (2)	0.43304 (16)	0.0215 (4)	
C3	0.2742 (3)	0.6957 (2)	0.35451 (16)	0.0206 (4)	
H3	0.2898	0.7488	0.2683	0.025*	
C4	0.2773 (2)	0.5161 (2)	0.39858 (16)	0.0185 (4)	
C5	0.2560 (2)	0.2462 (2)	0.58662 (16)	0.0190 (4)	
H5	0.2745	0.1667	0.5374	0.023*	
C6	0.2339 (3)	0.1765 (2)	0.71344 (16)	0.0197 (4)	
C7	0.2029 (3)	0.2955 (2)	0.78447 (17)	0.0223 (4)	
H7	0.1848	0.2496	0.8712	0.027*	
C8	0.1980 (3)	0.4781 (2)	0.73103 (16)	0.0212 (4)	
H8	0.1781	0.5576	0.7803	0.025*	
C9	0.2226 (2)	0.5440 (2)	0.60513 (16)	0.0190 (4)	
C10	0.2516 (2)	0.4322 (2)	0.52983 (16)	0.0175 (4)	
C11	0.2405 (3)	−0.0239 (2)	0.77465 (16)	0.0231 (5)	
H11	0.2825	−0.0853	0.7093	0.028*	
C12	0.3913 (3)	−0.0852 (2)	0.86437 (18)	0.0292 (5)	
H12A	0.5230	−0.0542	0.8205	0.044*	
H12B	0.3927	−0.2152	0.9012	0.044*	
H12C	0.3546	−0.0253	0.9289	0.044*	
C13	0.0355 (3)	−0.0808 (2)	0.83985 (18)	0.0286 (5)	
H13A	−0.0161	−0.0113	0.8976	0.043*	
H13B	0.0457	−0.2087	0.8844	0.043*	
H13C	−0.0542	−0.0588	0.7792	0.043*	

### Atomic displacement parameters (Å^2^)

**Table:**

	*U*^11^	*U*^22^	*U*^33^	*U*^12^	*U*^13^	*U*^23^
O1	0.0290 (7)	0.0150 (7)	0.0213 (7)	−0.0018 (5)	−0.0036 (6)	−0.0053 (5)
O2	0.0367 (8)	0.0165 (7)	0.0283 (8)	−0.0043 (6)	−0.0056 (6)	−0.0040 (6)
N1	0.0438 (10)	0.0293 (9)	0.0185 (9)	−0.0163 (8)	−0.0034 (7)	−0.0065 (7)
N2	0.0261 (9)	0.0182 (8)	0.0198 (8)	−0.0039 (6)	−0.0012 (7)	−0.0014 (6)
N3	0.0430 (11)	0.0413 (11)	0.0214 (9)	−0.0155 (8)	−0.0077 (8)	−0.0046 (8)
C1	0.0241 (9)	0.0201 (9)	0.0164 (9)	−0.0060 (7)	−0.0017 (7)	−0.0017 (7)
C2	0.0219 (9)	0.0200 (10)	0.0217 (10)	−0.0025 (7)	−0.0040 (7)	−0.0038 (8)
C3	0.0210 (9)	0.0211 (9)	0.0187 (9)	−0.0045 (7)	−0.0032 (7)	−0.0031 (8)
C4	0.0142 (8)	0.0213 (9)	0.0200 (9)	−0.0023 (7)	−0.0033 (7)	−0.0051 (7)
C5	0.0184 (9)	0.0196 (9)	0.0200 (9)	−0.0016 (7)	−0.0027 (7)	−0.0075 (8)
C6	0.0203 (9)	0.0180 (9)	0.0205 (9)	−0.0031 (7)	−0.0032 (7)	−0.0044 (7)
C7	0.0238 (9)	0.0243 (10)	0.0181 (9)	−0.0041 (7)	−0.0030 (7)	−0.0042 (8)
C8	0.0235 (9)	0.0208 (9)	0.0218 (10)	−0.0008 (7)	−0.0026 (8)	−0.0105 (8)
C9	0.0185 (9)	0.0151 (9)	0.0227 (10)	−0.0011 (7)	−0.0037 (7)	−0.0040 (7)
C10	0.0148 (8)	0.0191 (9)	0.0192 (9)	−0.0025 (7)	−0.0028 (7)	−0.0057 (7)
C11	0.0298 (10)	0.0193 (9)	0.0189 (10)	−0.0029 (8)	−0.0013 (8)	−0.0046 (7)
C12	0.0289 (10)	0.0215 (10)	0.0317 (11)	−0.0016 (8)	−0.0049 (9)	0.0004 (8)
C13	0.0334 (11)	0.0213 (10)	0.0305 (11)	−0.0061 (8)	−0.0070 (9)	−0.0036 (8)

### Geometric parameters (Å, º)

**Table:**

O1—C2	1.382 (2)	C6—C7	1.398 (3)
O1—C9	1.386 (2)	C6—C11	1.515 (2)
O2—C2	1.207 (2)	C7—C8	1.378 (2)
N1—N2	1.228 (2)	C7—H7	0.9500
N1—C1	1.471 (2)	C8—C9	1.378 (3)
N2—N3	1.133 (2)	C8—H8	0.9500
C1—C4	1.508 (2)	C9—C10	1.391 (3)
C1—H1A	0.9900	C11—C12	1.531 (3)
C1—H1B	0.9900	C11—C13	1.532 (3)
C2—C3	1.442 (3)	C11—H11	1.0000
C3—C4	1.346 (2)	C12—H12A	0.9800
C3—H3	0.9500	C12—H12B	0.9800
C4—C10	1.450 (2)	C12—H12C	0.9800
C5—C6	1.391 (2)	C13—H13A	0.9800
C5—C10	1.407 (2)	C13—H13B	0.9800
C5—H5	0.9500	C13—H13C	0.9800
			
C2—O1—C9	121.36 (14)	C7—C8—C9	119.10 (17)
N2—N1—C1	116.42 (14)	C7—C8—H8	120.5
N3—N2—N1	171.49 (17)	C9—C8—H8	120.5
N1—C1—C4	109.91 (14)	C8—C9—O1	115.88 (16)
N1—C1—H1A	109.7	C8—C9—C10	122.21 (16)
C4—C1—H1A	109.7	O1—C9—C10	121.90 (16)
N1—C1—H1B	109.7	C9—C10—C5	117.60 (16)
C4—C1—H1B	109.7	C9—C10—C4	117.48 (15)
H1A—C1—H1B	108.2	C5—C10—C4	124.92 (16)
O2—C2—O1	116.82 (16)	C6—C11—C12	111.82 (15)
O2—C2—C3	126.20 (17)	C6—C11—C13	111.03 (15)
O1—C2—C3	116.98 (15)	C12—C11—C13	110.35 (15)
C4—C3—C2	122.54 (17)	C6—C11—H11	107.8
C4—C3—H3	118.7	C12—C11—H11	107.8
C2—C3—H3	118.7	C13—C11—H11	107.8
C3—C4—C10	119.70 (16)	C11—C12—H12A	109.5
C3—C4—C1	121.56 (16)	C11—C12—H12B	109.5
C10—C4—C1	118.72 (15)	H12A—C12—H12B	109.5
C6—C5—C10	121.25 (16)	C11—C12—H12C	109.5
C6—C5—H5	119.4	H12A—C12—H12C	109.5
C10—C5—H5	119.4	H12B—C12—H12C	109.5
C5—C6—C7	118.58 (16)	C11—C13—H13A	109.5
C5—C6—C11	121.29 (16)	C11—C13—H13B	109.5
C7—C6—C11	120.13 (16)	H13A—C13—H13B	109.5
C8—C7—C6	121.25 (17)	C11—C13—H13C	109.5
C8—C7—H7	119.4	H13A—C13—H13C	109.5
C6—C7—H7	119.4	H13B—C13—H13C	109.5
			
N2—N1—C1—C4	−140.65 (17)	C2—O1—C9—C8	178.30 (14)
C9—O1—C2—O2	−177.52 (15)	C2—O1—C9—C10	−0.6 (2)
C9—O1—C2—C3	1.9 (2)	C8—C9—C10—C5	−0.2 (3)
O2—C2—C3—C4	177.40 (17)	O1—C9—C10—C5	178.68 (14)
O1—C2—C3—C4	−1.9 (3)	C8—C9—C10—C4	−179.50 (15)
C2—C3—C4—C10	0.7 (3)	O1—C9—C10—C4	−0.6 (2)
C2—C3—C4—C1	−178.12 (15)	C6—C5—C10—C9	−0.5 (3)
N1—C1—C4—C3	5.5 (2)	C6—C5—C10—C4	178.70 (15)
N1—C1—C4—C10	−173.34 (14)	C3—C4—C10—C9	0.6 (2)
C10—C5—C6—C7	1.3 (3)	C1—C4—C10—C9	179.43 (15)
C10—C5—C6—C11	−179.43 (15)	C3—C4—C10—C5	−178.67 (16)
C5—C6—C7—C8	−1.3 (3)	C1—C4—C10—C5	0.2 (2)
C11—C6—C7—C8	179.40 (15)	C5—C6—C11—C12	126.86 (18)
C6—C7—C8—C9	0.6 (3)	C7—C6—C11—C12	−53.9 (2)
C7—C8—C9—O1	−178.76 (14)	C5—C6—C11—C13	−109.42 (19)
C7—C8—C9—C10	0.2 (3)	C7—C6—C11—C13	69.9 (2)

### Hydrogen-bond geometry (Å, º)

**Table:**

*D*—H···*A*	*D*—H	H···*A*	*D*···*A*	*D*—H···*A*
C5—H5···O2^i^	0.95	2.56	3.498 (2)	168
C13—H13*C*···O2^ii^	0.98	2.55	3.524 (3)	172

Symmetry codes: (i) *x*, *y*−1, *z*; (ii) −*x*, −*y*+1, −*z*+1.
