# Supplementary figures and images for: Crystal structure of 4-azido­methyl-6-isopropyl-2H-chromen-2-one
Source: Acta Crystallogr E Crystallogr Commun. 2015 Mar 7;71(Pt 4):o227–8. doi: 10.1107/S2056989015004387 (PMC4438796; doi:10.1107/S2056989015004387)

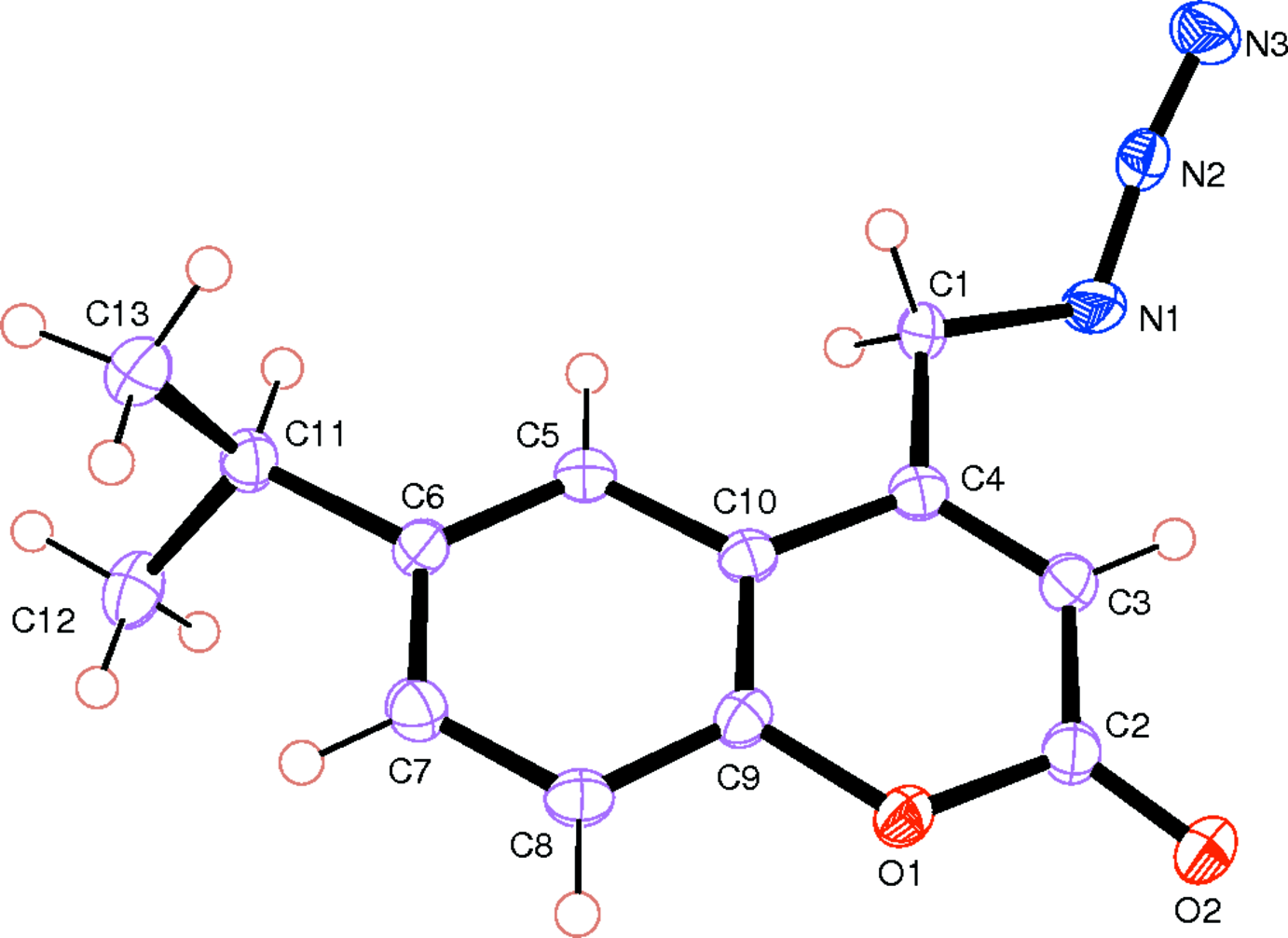

Supplement: Supplementary file 4 [file e-71-0o227-fig1.tif]

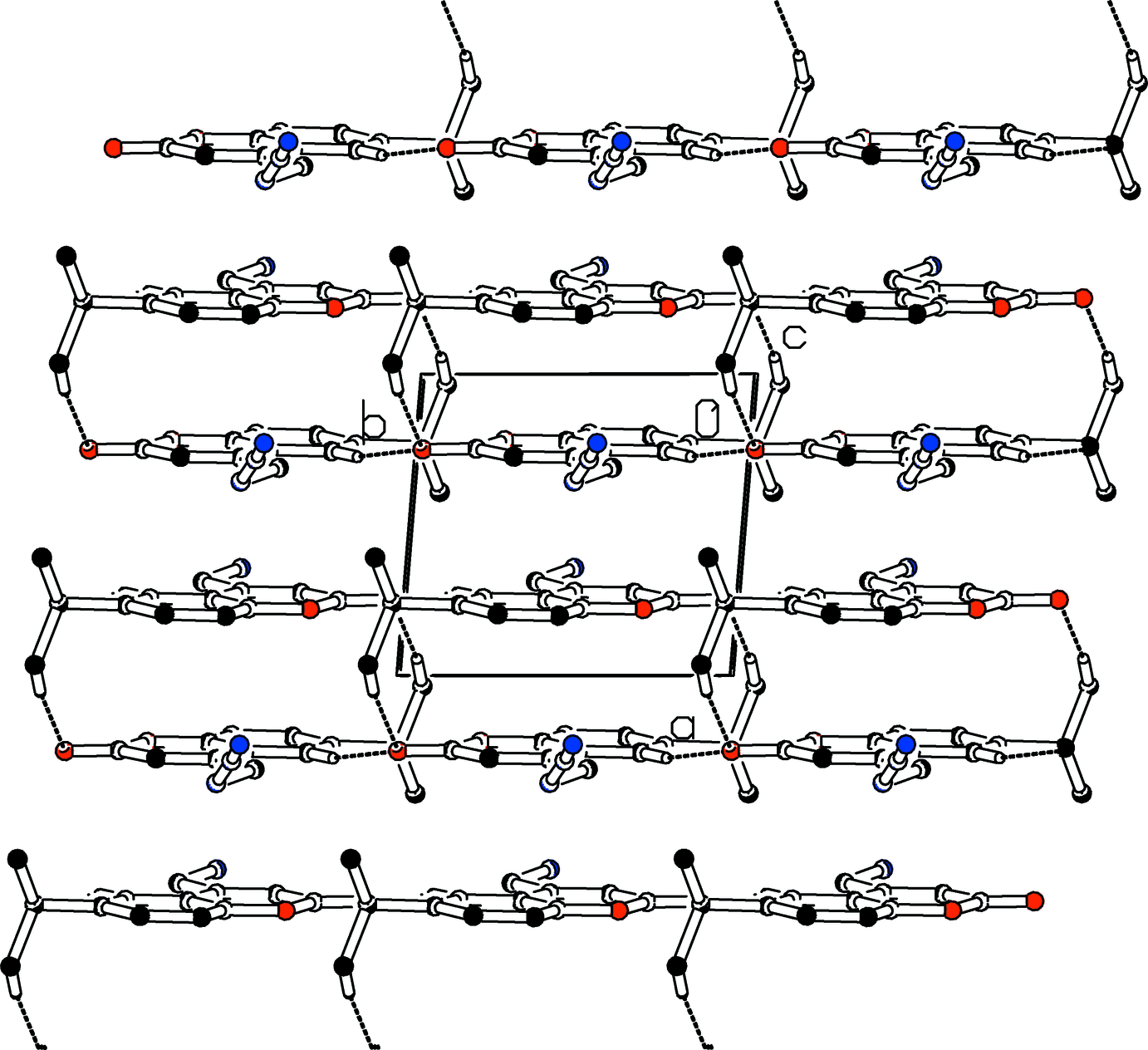

Supplement: Supplementary file 5 [file e-71-0o227-fig2.tif]
